# The Metric System

**Published:** 1880-07

**Authors:** 


THE METRIC SYSTEM.—We have been considerably surprised to find in allusions to this subject, occasionally, advocacy of a change to it from our old and familiar signs and symbols, weights and measures; but for the life of us we have been unable to see what advantage such a change could be to us in this country! This may be owing to our obtuseness of thought and intellect, but we are free to confess, and place the same upon record, that thus far we have been unable to adumbrate any thing concerning the proposed change that should cause us to be willing to substitute the metric for the old apothecaries weights and measures with which all English speaking and writing people are and have been so long familiar. In fact, we think the proposed change would be likely to do more harm than good amongst us, as it would doubtless lead to the commission of error in the writing and interpretation of physicians' prescriptions by druggists much more frequently than occurs under our present arrangement; and as it is not any more concise, elegant, or easy of comprehension, these are certainly very potent reasons for adhering to our existing system; but they are all too conspicuous to require notice from us, and we shall hold fast to that which is good, while we may continue to indite ℞'s for the interpretation and compounding by the apothecary.

